# I’ll emulate you…only if you want me to: The impact of ingroup norms and status on the identification-assimilation relationship

**DOI:** 10.1371/journal.pone.0195254

**Published:** 2018-04-25

**Authors:** Vincenzo Iacoviello, Fabio Lorenzi-Cioldi, Clara Kulich

**Affiliations:** 1 Social Psychology, Faculty of Behavioural and Social Sciences, University of Groningen, Groningen, the Netherlands; 2 Social Psychology, Section of Psychology, Faculty of Psychology and Educational Sciences, University of Geneva, Geneva, Switzerland; University of Jyväskylä, FINLAND

## Abstract

High identifiers are generally more willing to affiliate to their group and, as a result, perceive themselves and behave as prototypical members of their group. But is this always the case? The present research investigates the when and the why of the positive relationship between ingroup identification and assimilation by focusing on the role of the content of the injunctive ingroup norm (collectivistic vs. individualistic) and the ingroup status. Two experiments showed a positive identification-assimilation relationship in the low-status group when the ingroup norm was collectivistic, but not when the norm was individualistic. Moreover, the relationship was unreliable in the high-status group, regardless of the content of the norm. In a third study, these findings were extended to a more general measure of group affiliation (i.e., the need to belong). This research suggests that the greater tendency of high identifiers to assimilate to their group–and, more generally, to affiliate to groups–is accounted for by conformity motivations and strategies aimed at coping with an unfavorable social identity.

## Introduction

For a long time, social psychologists have investigated how group membership shapes the group members’ perceptions and behaviors. Typically, ingroup identification is associated with the willingness to affiliate with the group and to be committed to it [1]. In turn, motivation to affiliate to the group often results in ingroup assimilation (i.e., being close to the ingroup prototype) [2; 3]. High identifiers indeed tend to conform to the ingroup *descriptive* norm, that is, to “emulate” the ingroup’s typical behaviors [4]. However, conformity is not only related to what other people do (i.e., descriptive norms), but also to what other people encourage us to do (i.e., injunctive norms; see [5] for a further elaboration of this distinction). In other words, high identifiers are motivated to assimilate to the ingroup (i.e., to conform to the descriptive norm), as well as to stick to the ingroup’s normative guidelines (i.e., to conform to the injunctive norm, see [6]).

Indeed, research suggests that the relationship between ingroup identification and assimilation is contingent upon the content of the ingroup injunctive norm [7; 8; 9]. When the ingroup norm promotes collectivism (i.e., when it stresses interpersonal solidarity and group belonging), ingroup identification calls for self-others assimilation. The high-identifiers’ motivation to come close to the ingroup prototype and to endorse the ingroup norm act concurrently. Conversely, when the ingroup norm promotes individualism (i.e., when it stresses autonomy and personal uniqueness), the identification-assimilation relationship is of weaker magnitude. In such instance, the high-identifiers’ motivation to assimilate to the ingroup diverges from their motivation to follow an ingroup norm that encourages the quest for individual uniqueness. The genuine tendency to assimilate to the ingroup prototype is then at odds with the tendency to stress personal uniqueness. This strain has been voiced by [10] when they contended that "conformity to individualist group norms will enhance intragroup differentiation and increase diversity, which goes against the tendency for uniformity encouraged by social identity salience. The consequence of this is that individualism in a group context is more likely to be moderate than extreme because the salience of the group provides a counterforce to greater cohesion and uniformity" (p.124). In sum, the positive identification-assimilation relationship is weaker when the ingroup norm is individualistic, as compared to when the ingroup norm is collectivistic. The present work seeks to deepen our knowledge of this process by considering a further moderator of the identification-assimilation relationship: ingroup status.

The literature documents that the relationship between ingroup identification and assimilation is also contingent on the social identity’s value (e.g., [11]). Group status, that is, the rank ordering of groups on consensually valued dimensions, is a major determinant of the identity’s value [12]. Generally, members of high-status groups benefit from a satisfying social identity, while members of low-status groups experience threats to their social identity. In order to attain self-worth, the latter use a variety of strategies, depending on their level of ingroup identification [13]. Those for whom the ingroup is crucial for defining the self (high identifiers) tend to assimilate to the ingroup. Ingroup assimilation fosters feelings of community and collective support among the group members, and improves efficacy in reaching their group goals [14; 15; 16]. Conversely, low identifiers tend to distance themselves from the ingroup to get rid of the unsatisfactory group membership [17]. This difference between the two assimilation tendencies is more relevant for members of low-status groups (who are highly motivated to restore self-worth) than for members of high-status groups (to whom a positive social identity is granted). In support of this rationale, research has shown that, in low-status groups, identification is positively associated with self-stereotyping [11], perception of ingroup homogeneity [18], and collectivistic worldviews [19], while these associations are weaker in high-status groups. Accordingly, we expect high-identified members of a low-status group, compared to the low identified, not only to assimilate more strongly to the ingroup, but also to be more responsive to the ingroup norm (since both ingroup assimilation and conformity to the norm strengthen the bonds between self and ingroup [20]).

We thus hypothesize that the identification-assimilation relationship in the low-status group should be positive when the ingroup norm is collectivistic, while this relationship should fade away when the norm is individualistic. In this latter case, high identifiers should indeed experience a conflict between their genuine tendency to assimilate to the ingroup and their tendency to follow the individualistic norm which promotes personal distinctiveness. Conversely, in the high-status group, the identification-assimilation relationship should be weak or null regardless of the content of the norm. Given that the value of the social identity is granted, high and low identifiers should not be differently motivated to assimilate to the ingroup and to stick to the ingroup norm.

A study by [2] provides initial evidence in favor of this conjecture. The authors examined the relationship between ingroup identification and assimilation (as measured by self-stereotyping) as a function of ingroup norm (collectivistic vs. individualistic) and identity threat (present vs. absent). The identity threat manipulation, a proxy of ingroup status, consisted of comparing the participants’ ingroup (i.e., their home university) either favorably or unfavorably to the national average on many criteria (including for instance the students’ prospect of future career). The findings revealed the predicted norm moderation of the relationship between identification and self-stereotyping in the threat condition, but not in the no-threat condition. However, in this study, we could argue that there was a confound between the threat to the social identity’s value as indicated by the relative prestige of the ingroup (i.e., ingroup status), and the threat to the personal self (as indicated by the prospect of their future career). Indeed, it is possible that high identifiers in the threat condition followed the collectivistic norm because they were negatively affected at a personal level (i.e., by the pessimistic prospect of their future career), and not because of the threat on the value of their ingroup as a whole.

The present research examined more straightforwardly the role of group status on the identification-assimilation relationship. Moreover, we moved a step further from the above-mentioned research, by arguing that in the low-status group, the high-identifiers’ strategy to cope with a threat to their social identity is not confined to ingroup assimilation, but encompasses a more general sense of belonging to social groups. Accordingly, the high-identified members’ conformity to the low-status group’s collectivistic norm would translate into a general inclination toward collectivism, and thus to an increased willingness to tighten bonds with social groups.

## Current research

The present research aims at investigating the combined impact of the content of the ingroup norm and ingroup status on the relationship between ingroup identification and assimilation. Since ingroup assimilation is the result of affiliation motives [2; 3], we will further extend this investigation on a more general indicator of group affiliation, that is, the need to belong to social groups. In three studies, ingroup status was created by priming a favorable or unfavorable comparison of the participants’ home university to a rival university. The content of the norm (collectivistic vs. individualistic) was either primed as a generic norm (Study 1) or as a specific ingroup norm (Studies 2–3). In Studies 1–2, ingroup assimilation was assessed through the endorsement of typical ingroup behaviors (i.e., regarding the choice of a product). In Study 3, we assessed participants’ motive for a general group affiliation. The hypothesis of the present studies is that in the low-status group the positive relationship between identification and assimilation (or group affiliation) should be more pronounced when the norm is collectivistic than when the norm is individualistic. Conversely, in the high-status group the corresponding relationship should be weak or null, regardless of the content of the ingroup norm. In each study, participants were students from the University of Geneva. They were contacted by email and were asked to participate in an online study. We aimed at recruiting 40–50 participants per experimental cell [21]. Because the studies’ design was a 2 (norm) × 2 (status), we targeted about 160 to 200 participants per study.

These studies did not involve medical or health related experimentation. Each study was approved by the Research Ethics Committee of the Faculty of Psychology at the University of Geneva, Switzerland. The participation was voluntary. Participants’ consent was obtained when they clicked on the link to start the online survey and at the end of the survey, after having been fully debriefed about the purpose of the study.

## Study 1

In Study 1, we measured ingroup identification and manipulated ingroup status and the content of a generic norm. The main dependent variable consisted of the assessment of ingroup assimilation.

### Materials and method

#### Participants

One hundred and sixty students from the University of Geneva (115 women, 44 men, and 1 sex-unspecified, *M*_age_ = 24.29, *SD*_age_ = 5.78) completed an online questionnaire. The cover story introduced the study as measuring “the typical behaviors, preferences and characteristics of students from the University of Geneva”.

#### Procedure

Participants answered a six-item identification scale (1 = *not at all*, 7 = *completely*) [15]. Sample items are: “When I introduce myself, I like to mention that I am a student from the University of Geneva”, “When someone praises students from my university, it feels like a personal compliment”, and “Students of the University of Geneva should be proud of themselves” (α = .82, *M* = 3.59, *SD* = 1.20). They were then invited to perform a task aimed at priming a generic behavioral norm. The task was about either collectivism or individualism as praised ways of being. In the collectivistic norm condition (vs. individualistic norm condition in brackets), participants were given three minutes to think about “situations in which someone is admired when s/he acts in a united (vs. autonomous) way” and to report these situations in a box below. Participants were then presented with a fictitious newspaper article aiming at manipulating ingroup status (see [15]). Participants read an excerpt of a fictitious newspaper article about the rank order of universities around the world. The article explained that an organization named *Ranking of World Universities* had rated universities on the basis of the quality of teaching and research, and of the students’ skills at the end of their university education. The excerpt focused on the comparison between the participants’ university and a rival nearby university (University of Y). In the high-status condition, participants learned that their university was superior to the outgroup university. This information was illustrated by a graph showing that, in the course of time, the University of Geneva had received better scores than the University of Lausanne. In the low-status condition, participants were given the reverse information, with University of Geneva being depicted as inferior to University of Lausanne.

Assimilation to the ingroup was the dependent variable. It was assessed through the choice of a computer game that was typically preferred (or not) by ingroup members. Participants were asked to choose a computer game among four different games, and were informed that they would play the chosen game after completion of the experiment. Each game was portrayed with a picture and a short description sentence. The sole critical difference between games consisted in the proportion of ingroup students who had previously chosen each game. Participants learned that a majority (41% and 42%) had chosen two games while a minority (8% and 9%) had chosen the two other games. The order in which the games were presented was randomized across participants. In order to provide a check of the effectiveness of the status manipulation, participants were asked to think of the excerpt they had read earlier and to indicate their mood. Four positive affects (enthusiastic, proud, calm, and satisfied) and four negative affects (annoyed, irritated, sad, and worried) were proposed (1 = *not at all*, 7 = *completely*). We computed two mood scores by averaging the four positive (α = .75, *M* = 3.79, *SD* = 1.32) and the four negative affects (α = .77, *M* = 2.16, *SD* = 1.25). The final mood score consisted of the subtraction of the negative mood score from the positive mood score (*M* = 1.63, *SD* = 2.02). Thus, a positive difference score indicates a positive mood and a negative difference score a negative mood. We expect the mood score to be greater in the high-status than in the low-status condition, and this status discrepancy to be of greater magnitude among highly-identified participants. At the end of the survey, participants provided their demographics and were fully debriefed.

### Results

#### Mood

We performed a linear regression analysis on the mood score with norm (coded -1 for collectivistic, and 1 for individualistic), status (coded -1 for low, and 1 for high), ingroup identification (centered continuous variable) and their interactions as predictors. The analysis first revealed a main effect of status, *B* = 0.69, *t*(152) = 4.64, *p* < .001, 95% CI [0.40, 0.98], corroborating that the mood score was greater in the high-status condition than in the low-status condition (*Ms* = 2.25 and 0.88, respectively). As expected, this effect was qualified by a Status × Identification interaction, *B* = 0.44, *t*(152) = 3.46, *p* = .001, 95% CI [0.19, 0.69]. In the high-status condition, the mood score increased as a function of ingroup identification (*Ms* = 1.68 and 2.83, for low [-1 SD] and high identifiers [+1 SD] respectively), *B* = 0.48, *t*(152) = 2.76, *p* = .007, 95% CI [0.14, 0.83]. Conversely, in the low-status condition, the mood score decreased as a function of ingroup identification (*Ms* = 1.34 and 0.41, for low [-1 SD] and high identifiers [+1 SD] respectively), *B* = -0.39, *t*(152) = -2.15, *p* = .03, 95% CI [-0.75, -0.03]. No other effects were significant, all *ps* > .20.

#### Ingroup assimilation

Analysis of game choice first revealed that majority games were selected less often than minority games (33.1% and 66.9%, respectively), *χ*^*2*^ (1, N = 160) = 18.23, *p* < .001. In order to test for our hypothesis—that the positive identification-assimilation relationship should be particularly conspicuous in the low-status group when the norm is collectivistic—we first computed three Helmert contrasts combining the norm and status variables. The first contrast (C1) opposed the low-status/collectivistic norm condition (coded 3) to the other three conditions (all coded -1). The second contrast (C2) opposed the low-status/individualistic norm condition (coded 2) to both the high-status/collectivistic norm and the high-status/individualistic norm conditions (both coded -1). The third contrast (C3) compared the high-status/collectivistic norm condition (coded 1) to the high-status/individualistic norm condition (coded -1). Our hypothesis predicts an interaction between C1 and ingroup identification. C2 and C3 are residual contrasts and we do not have specific hypotheses about them.

**Fig 1:**
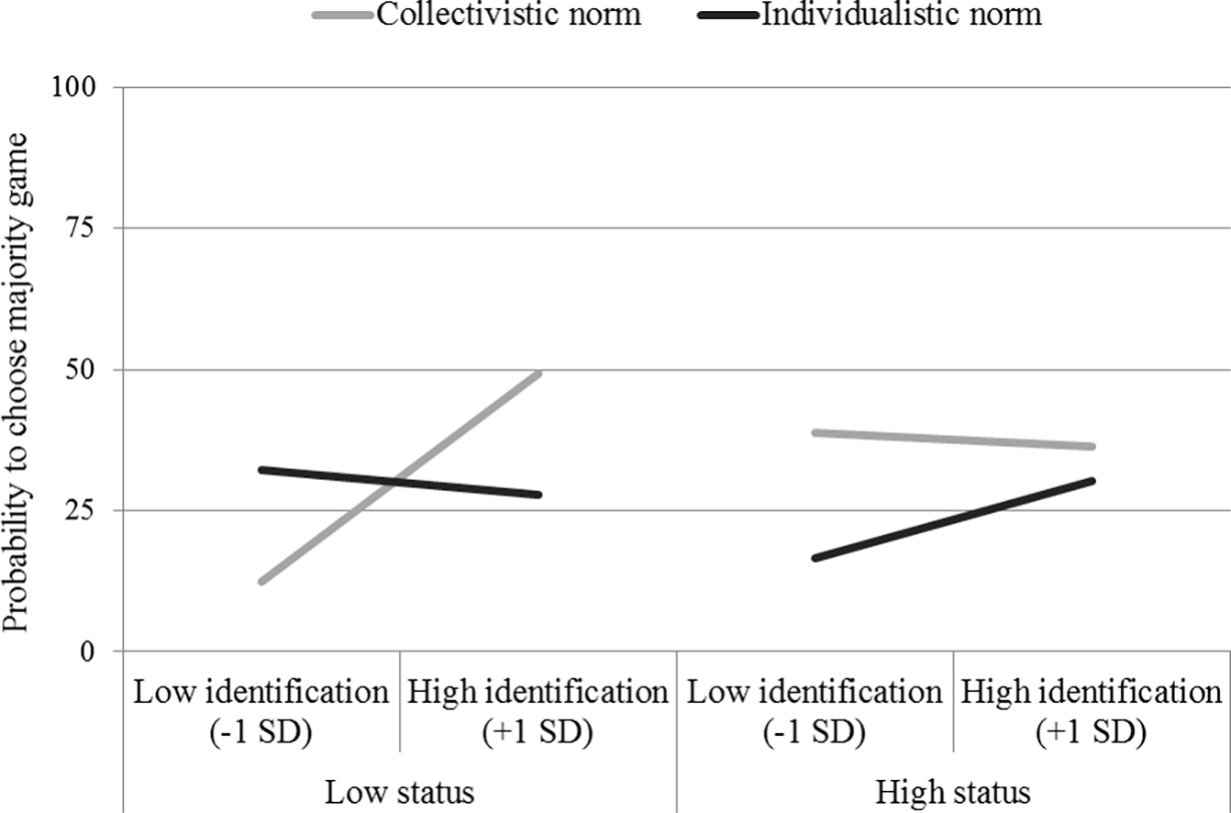
Majority game choice according to ingroup identification, primed norm and ingroup status (Study 1).

We performed a logistic regression on game choice (minority = 0, majority = 1) with the predictors consisting of the three contrasts (C1, C2 and C3), ingroup identification (centered continuous variable), and the interactions between each contrast and ingroup identification. In line with expectation, the analysis produced a significant C1 × Identification interaction, *B* = 0.24, *χ*^*2*^ (1, N = 160) = 5.30, *p* = .02, *e*^*B*^ = 1.27, suggesting that the identification-assimilation relationship in the low-status/collectivistic norm condition differed from the other three conditions. As is apparent from Fig 1, this relationship was indeed positive and significant in the low-status/collectivistic norm condition, *B* = 0.96, *χ*^*2*^ (1, N = 160) = 6.46, *p* = .01, *e*^*B*^ = 2.62. Conversely, in the other three conditions, the relationship was not reliable (in the low-status/individualistic norm condition, *B* = -0.10, *χ*^*2*^ (1, N = 160) = 0.09, *p* = .76, *e*^*B*^ = 0.90; in the high-status/collectivistic norm condition, *B* = -0.29, *χ*^*2*^ (1, N = 160) = 1.00, *p* = .32, *e*^*B*^ = 0.75, in the high-status/individualistic norm condition, *B* = 0.39, *χ*^*2*^ (1, N = 160) = 1.55, *p* = .21, *e*^*B*^ = 1.47).

The logistic regression also showed an unexpected main effect of C3. Among participants in the high-status condition, the likelihood of choosing a majority game was higher in the collectivistic than in the individualistic norm condition (49.2% and 22.9%, respectively), *B* = 0.59, *χ*^*2*^ (1, N = 160) = 5.29, *p* = .02, *e*^*B*^ = 1.81. All other effects (including the C2 × Identification and the C3 × Identification interactions) were not statistically significant, all *ps* > .11.

### Discussion

Testifying to the effectiveness of the status manipulation, and replicating previous research (i.e., [19]), the present study showed that participants in the high-status condition reported more positive mood than those in the low-status condition, and that this status difference increased as a function of ingroup identification. Since these findings provide firm evidence for the arousing affective impact of the status manipulation, the following studies did not include this measure and focused on ingroup assimilation and group affiliation as dependent variables. The findings on the main dependent variable (i.e., ingroup assimilation) first showed a general tendency to choose minority games (over majority games). This tendency reveals congruent with the Western cultural penchant for individualism and personal uniqueness [22; 23]. The findings then provided support for our hypothesis by showing that, in the low-status condition, the positive relationship between identification and assimilation to the ingroup only appeared when the norm was collectivistic. In the high-status condition, assimilation to the ingroup was unrelated to identification, whatever the ingroup norm. The findings also unexpectedly revealed that, in the high-status condition, participants primed with the collectivistic norm exhibited greater ingroup assimilation than those primed with the individualistic norm. Participants in the high-status condition seem to have been influenced by the primed generic norm, but this effect revealed unrelated to their level of ingroup identification. The same pattern was not observed in the low-status group because, as predicted, low identifiers primed with a collectivistic norm acted at odds with the norm. Future studies will indicate whether this effect is reliable or not.

In the present study, the norm manipulation revolved around ways of being which are normatively prescribed [24]. A limitation of this procedure is that the prime did not explicitly refer to the ingroup. The following studies thereby manipulated the content of the ingroup norm by referring explicitly to the participant membership group.

## Study 2

This study aimed at replicating the findings from Study 1 with a manipulation of the norm which was more explicitly anchored in the ingroup.

### Materials and method

#### Participants

We contacted students from the University of Geneva by e-mail and asked them to participate in an online questionnaire with the same cover story as in Study 1. A total of 195 students (133 women, 62 men, *M*_age_ = 22.79, *SD*_age_ = 5.30) completed the questionnaire.

#### Procedure

Participants first answered the same ingroup identification scale as in Study 1 (α = .83, *M* = 3.95, *SD* = 1.14), and read the fictitious article aimed at inducing ingroup status. Afterwards, ingroup norm was induced by informing participants about the results of a previous study conducted with other ingroup students on opinions about advertisement messages. The alleged results delivered the mean persuasiveness ratings of four collectivistic and four individualistic advertising messages. We had previously conducted a pilot study in order to select these messages, in which we asked 20 students to rate the extent to which each of a sample of advertisement messages emphasized differences or similarities among people (1 = *Differences*, 7 = *Similarities*). We selected four messages that loaded substantially toward the similarity pole (α = .70, *M* = 4.88, *SD* = 1.07; difference from the scale midpoint, *t*(19) = 3.66, *p* < .01, *d* = 1.68) and four messages that loaded substantially toward the difference pole (α = .72, *M* = 2.83, *SD* = 0.92; difference from the scale midpoint, *t*(19) = -5.72, *p* < .001, *d* = -2.62). Sample collectivistic messages are “Together, it’s better” and “Everybody benefits”. Sample individualistic messages are “Find your true nature” and “Open your own path in the crowd”.

**Fig 2:**
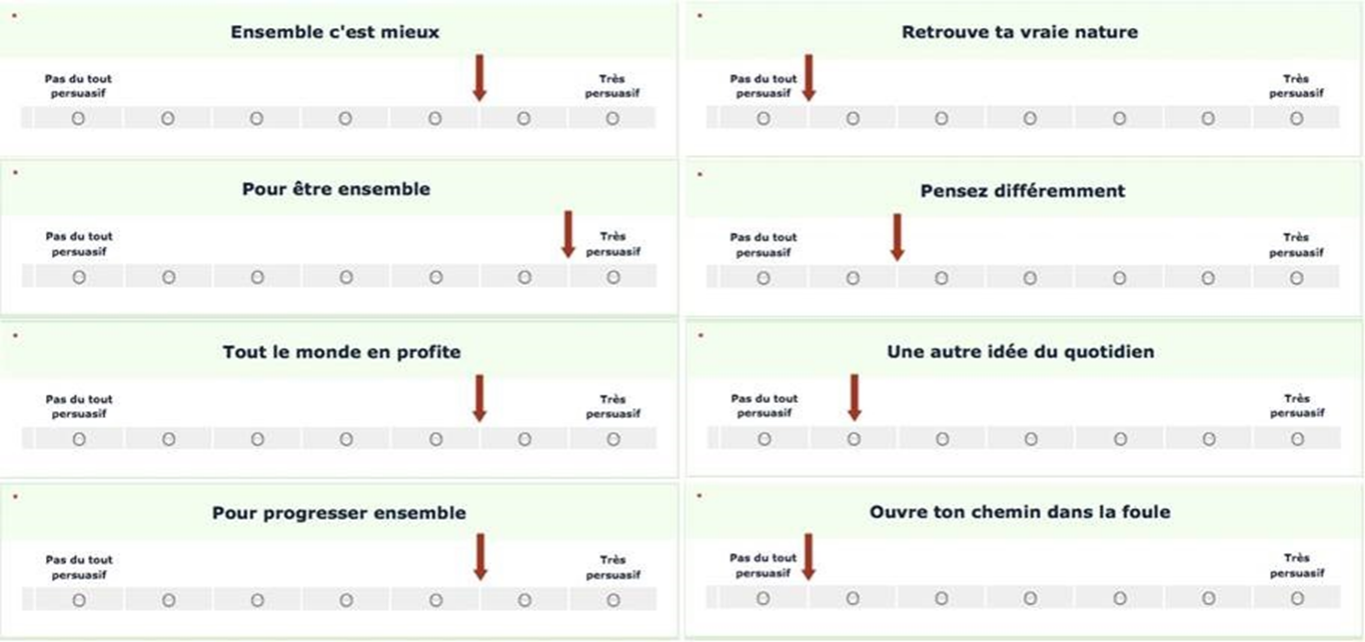
The messages used in Studies 2–3 for the induction of the collectivistic ingroup norm. On each message, the red arrow indicates the mean ratings of ingroup university students in the alleged previous study. In this condition, collectivistic messages (left panel) had high means, whereas individualistic messages (right panel) had low means (in the individualistic condition, the pattern of means was reversed). The original messages were presented in French. Hereafter, we provide the English translation for each type of messages (from top to bottom). Collectivistic messages: “Together, it’s better”, “To be together”, “Everybody benefits” and “To move forward together”. Individualistic messages: “Find your true nature”, “Think differently”, “Another idea of everyday life” and “Open your own path in the crowd”.

In the main study, participants were presented with these messages, listed in a random order, and their 7-point Likert scales (1 = *not at all persuasive*, 7 = *very persuasive*). On each message’s scale, a red arrow indicated the mean ratings of the ingroup university students from the alleged previous study. In the collectivistic norm condition, the persuasiveness ratings of the four collectivistic messages were ostensibly high and the persuasiveness ratings of the four individualistic messages were ostensibly low (see Fig 2). The reverse pattern was presented to participants in the individualistic norm condition. Similar to Study 1, participants were then asked to choose a computer game, which was our measure of ingroup assimilation. Finally, they reported demographics and were debriefed about the purpose of the study.

### Results

**Fig 3:**
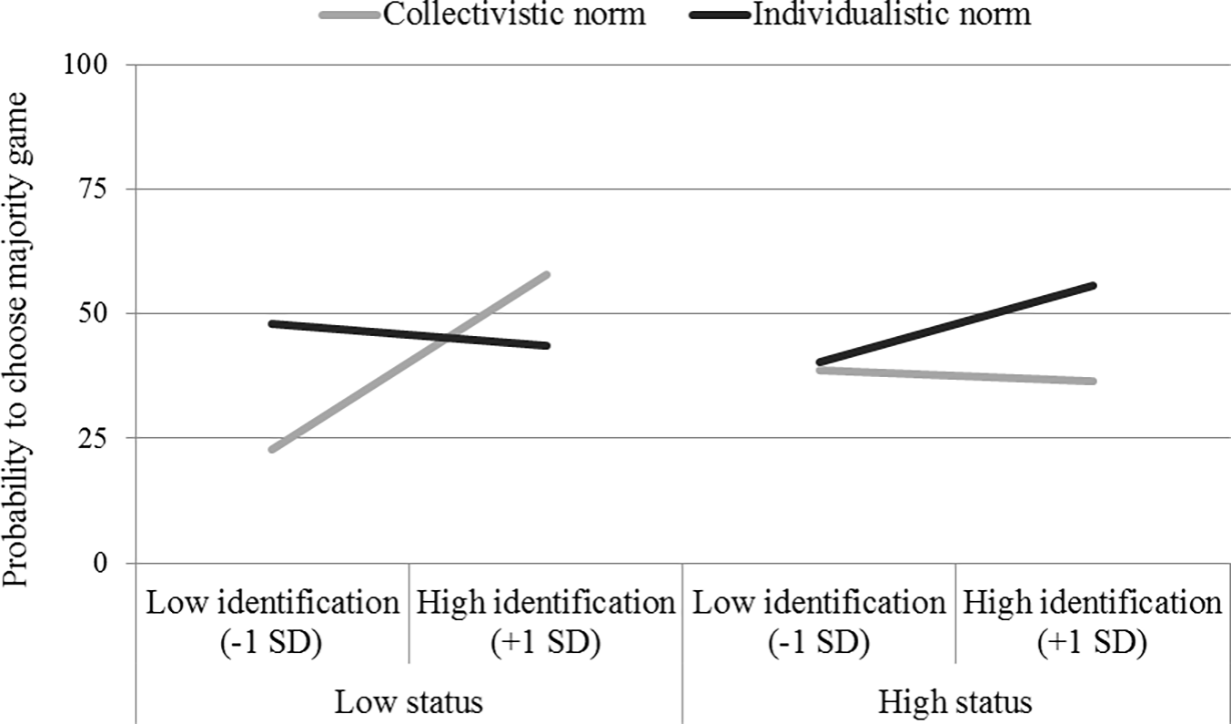
Majority game choice according to ingroup identification, norm and status (Study 2).

A χ^2^ analysis showed, as in Study 1, that minority games tended to be chosen more often than majority games (56.4% and 43.6%, respectively), *χ*^*2*^ (1, N = 195) = 3.21, *p =* .07. In order to test for our hypothesis, we computed the same Helmert contrasts as in Study 1, and we performed a logistic regression on game choice (minority = 0, majority = 1) with the three contrasts (C1, C2 and C3), ingroup identification (centered continuous variable), and the interactions between each contrast and identification as predictors. The analysis first revealed a marginally significant main effect of ingroup identification, which indicated a positive relationship between ingroup identification and the likelihood of choosing a majority game, *B* = 0.24, *χ*^*2*^ (1, N = 195) = 2.93, *p* = .09, *e*^*B*^ = 1.27. Consistent with our hypothesis, this main effect was qualified by a C1 × Identification interaction, *B* = 0.18, *χ*^*2*^ (1, N = 195) = 3.77, *p* = .05, *e*^*B*^ = 1.20. The identification-assimilation relationship was more conspicuous in the low-status/collectivistic condition than in the other three conditions (see Fig 3). Inspection of simple slopes indeed showed that, in the low-status/collectivistic condition, ingroup identification and the choice of a majority game were positively related, *B* = 0.77, *χ*^*2*^ (1, N = 195) = 5.27, *p* = .02, *e*^*B*^ = 2.16. Conversely, ingroup identification was not significantly related to the likelihood of choosing a majority game in the other conditions (in the low-status/individualistic norm condition, *B* = -0.09, *χ*^*2*^ (1, N = 160) = 0.11, *p* = .74, *e*^*B*^ = 0.92; in the high-status/collectivistic norm condition, *B* = -0.05, *χ*^*2*^ (1, N = 160) = 0.04, *p* = .84, *e*^*B*^ = 0.95, in the high-status/individualistic norm condition, *B* = 0.31, *χ*^*2*^ (1, N = 160) = 1.36, *p* = .24, *e*^*B*^ = 1.37). All other effects (including the C2 × Identification and the C3 × Identification interactions) were not reliable, all *ps* > .27.

### Discussion

Study 2’s findings again highlighted people’s tendency to emphasize personal uniqueness, showing an overall preference for minority games over majority games. More relevant for our hypothesis, the findings demonstrated a positive relationship between ingroup identification and assimilation only in the condition in which participants were assigned to a low-status group and the ingroup norm was collectivistic. This study thus replicated the results of Study 1 with a norm manipulation that referred more explicitly to the ingroup, supporting the idea that high-identified members of the low-status group are particularly keen to conform to the ingroup injunctive norm.

## Study 3

Study 3 used a similar design as Study 2. The main difference was the dependent variable. While Studies 1–2 focused on assimilation to the ingroup, the present study introduced a broader indicator of group affiliation (i.e., the need to belong).

### Materials and method

#### Participants

A total of 156 students of the University of Geneva (108 women, 45 men, 3 sex-unspecified; *M*_age_ = 25.72, *SD*_age_ = 8.72) completed the online questionnaire.

#### Procedure

Participants first answered three items assessing the centrality dimension of ingroup identification [25]. As stated by the authors of this multi-dimensional identification scale, centrality is the identification dimension that best predicts group members’ sensitivity to social identity threats (see also [26]). The items adapted for our study are: “I often think about the fact that I am a student of the University of Geneva”, “Being a student of the University of Geneva is an important part of my identity” and “Being a student of the University of Geneva is an important part of how I see myself” (α = .88, *M* = 3.68, *SD* = 1.56). We then manipulated ingroup status and ingroup norm as in Study 2. This was followed by the group affiliation dependent measure. We used the ten-item scale to assess the need to belong [27]. Sample items are: “I want other people to accept me”, “My feelings are easily hurt when I feel that others do not accept me (reverse-scored)” and “I have a strong ‘need to belong’”. After proper recoding, a need to belong score was computed such that high values indicate high levels of need to belong (α = .78, *M* = 4.33, *SD* = 0.98). At the end, participants provided their demographics and were fully debriefed.

### Results

**Fig 4:**
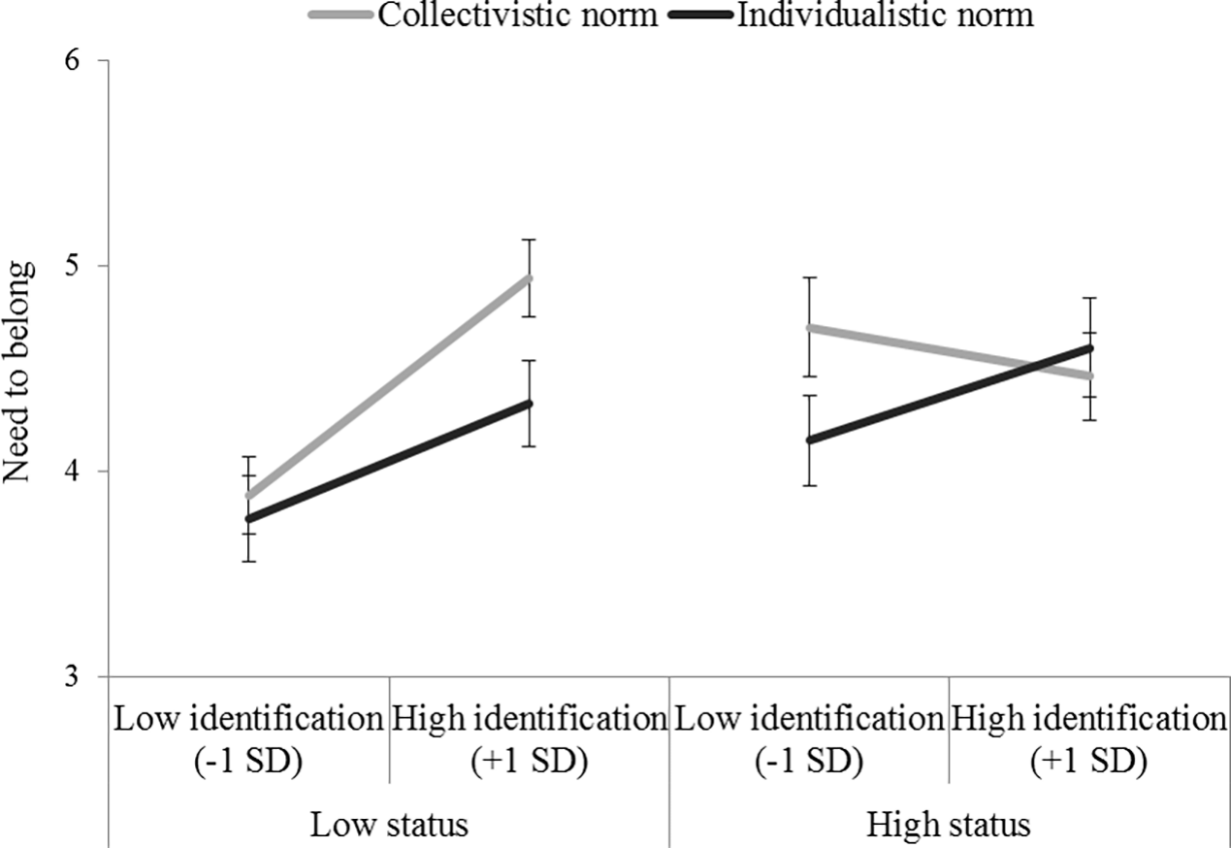
Means of need to belong according to ingroup identification, norm and status (Study 3). Error bars represents ±1 SE.

As in Studies 1–2, we computed the three Helmert contrasts. We then performed a linear regression on the need to belong score with the three contrasts (C1, C2 and C3), identification (centered continuous variable), and the interactions between each contrast and identification as predictors. The analysis produced a main effect of identification indicating that, overall, ingroup identification was positively related to the need to belong, *B* = 0.15, *t*(148) = 2.99, *p* = .003, 95% CI [0.05, 0.24]. The analysis also revealed the expected C1 × Identification interaction, *B* = 0.07, *t*(148) = 2.62, *p* = .01, 95% CI [0.16, 0.11]. The positive relationship between identification and need to belong was particularly conspicuous in the low-status condition when the ingroup norm promoted collectivism (see Fig 4). Simple slopes showed a positive and significant identification-belong relationship in the low-status/collectivistic norm condition, *B* = 0.34, *t*(148) = 4.33, *p* < .001, 95% CI [0.19, 0.50]. This relationship was only marginally significant in the low-status/individualistic norm condition, *B* = 0.18, *t*(148) = 1.86, *p* = .06, 95% CI [-0.01, 0.37], and was not significant in both the high-status/collectivistic norm condition, *B* = -0.08, *t*(148) = -0.76, *p* = .45, 95% CI [-0.28, 0.13], and the high-status/individualistic norm condition, *B* = 0.14, *t*(148) = 1.30, *p* = .20, 95% CI [-0.07, 0.36].

The analysis also produced an unexpected main effect of C2, *B* = -0.14, *t*(148) = -2.43, *p* = .02, 95% CI [-0.26, -0.03]. The need to belong score was lower in the low-status/individualistic norm condition (*M* = 4.05, *SE* = 0.14) than in the modality combining the high-status/collectivistic norm and the high-status/individualistic norm conditions (*M* = 4.48, *SE* = 0.11). All other effects (including the C2 × Identification and the C3 × Identification interactions) were not statistically significant, all *ps* > .14.

### Discussion

Study 3 extended the findings from the previous studies to a general measure of affiliation to social groups, commonly known as the need to belong. When exposed to a collectivistic ingroup norm, the highly identified low-status group members displayed a greater need to belong to social groups than the lowly identified. This tendency was weaker when the norm was individualistic as well as among members of the high-status group who were primed with either the collectivistic or the individualistic ingroup norm.

## General discussion

The present research examined the relationship between ingroup identification and ingroup assimilation (or group affiliation) as a function of ingroup status and content of the ingroup (injunctive) norm. Consistent with our hypothesis, high identifiers assimilated to the ingroup more than low identifiers, but this occurred only when the ingroup status was low and the norm was collectivistic. These findings corroborate the conjecture that in situations where individuals’ social identity is under threat (in this research, due to the unfavorable ingroup-outgroup comparison), high identifiers are motivated to cope with the threat by assimilating to the ingroup (i.e., conforming to the descriptive norm) and following its normative guidelines (i.e., conforming to the injunctive norm). Conversely, low identifiers show self-ingroup distancing and defy the group’s normative expectations.

When the norm prescribes collectivism, the tendencies to assimilate to the ingroup and to follow its norm act in concert for low-status group members. Thus, high levels of identification are straightforwardly associated with ingroup assimilation. However, when the norm prescribes individualism, the tendency to get close to the ingroup prototype is at odds with the tendency to follow the individualistic norm. In other words, high identifiers from low–status groups may feel unsure about whether to assimilate to the ingroup or to emphasize their personal distinctiveness (as promoted by the ingroup norm). Likewise, low identifiers may feel hesitant about whether distancing from the ingroup or violating the norm (by appearing less individualistic). These conflicts eventually produce a null relationship between identification and assimilation.

We confronted this situation of identity threat with a situation where this threat was absent because of the high status of the ingroup. We argued and found that in situations where the social identity is secure due to the consensually acknowledged favorable intergroup comparison, people have no need to cope with social identity threat, and thus no need to rely on assimilation (for high identifiers) or distancing (for low identifiers) strategies. As a result, the identification-assimilation relationship turns out to be null among members of the high-status group regardless of the collectivistic or individualistic content of the ingroup norm.

Finally, these same tendencies were observed on a more general indicator of group affiliation, that is, the need to belong. In situations where individuals' social identity is challenged and the group’s norm prescribes collectivism, high identifiers not only assimilate to their ingroup, but they also feel the desire to be more generally committed to social groups. This suggests that the endorsement of the ingroup collectivistic norm has far-reaching consequences and does not limit to ingroup assimilation.

### Theoretical considerations and future studies

This research examined the identification-assimilation relationship in groups experiencing (low status groups) or not (high status groups) social identity threat. Though informative, alternative interpretations of the discrepancy between the behaviors of the two status groups are left open. One may wonder whether the identification-assimilation relationship is triggered by the willingness to cope with the identity threat (as it is likely the case in the low-status group) or whether it is hindered by the secure social identity (as it is likely the case in the high-status group). Ongoing research that compares both status conditions to a condition in which the ingroup status is unspecified or unknown speaks in favor of the second explanation, but more studies are needed to resolve this point (see [28]).

Relatedly, we may ask: Are highly identified members of the high-status group unconditionally immune to the ingroup normative influence? We believe the answer is negative. In the present research, the ingroup status manipulation was likely to induce a perception of stability and legitimacy of the social hierarchy. These particular features of the hierarchy prompt feelings of security about the value of the members’ social identity. Hence, instability and/or illegitimacy of the favorable position of the ingroup in the hierarchy could trigger feelings of identity threat in the high-status group. High identifiers could therefore be tempted to use assimilation strategies (vs. distancing strategies, for low identifiers) in order to cope with this threat. Future studies should thus examine how perceptions of instability (vs. stability) and illegitimacy (vs. legitimacy) influence the identification-assimilation relationship among members of the high-status group.

Another important theoretical consideration pertains to the relative influence of both the motivation to assimilate to the ingroup prototype (i.e., conforming to the descriptive norm) and the motivation to follow the ingroup normative guidance (i.e., conforming to the injunctive norm). We have assumed that the null relationship between ingroup identification and assimilation in the low status/individualistic norm is due to these two motives cancelling each other out. This presupposes that both motives possess the same strength. However, it could be that in some circumstances one motive overcomes the other. Research indeed shows that injunctive norms are sometimes more influential than descriptive norms in shaping people’s behavior [29; 30]. For instance, when there is uncertainty about the self (e.g., who I am, how I should behave), one may expect the motivation to follow the ingroup guidelines to surpass the motivation to assimilate to the ingroup [31]. In this case, highly identified members of the low-status group would comply with the injunctive individualistic norm, and hence would distance themselves from the ingroup prototype in an apparent paradox.

## Conclusion

The present research shows that high identifiers are not unconditionally keen to “emulate” the ingroup’s typical behaviors. Rather, the relationship between ingroup identification and assimilation depends on complex dynamics related to ingroup status and the content of the ingroup norm. Assimilating the self to the ingroup prototype is thus not a mere cognitive process. It also relies on conformity to social expectations [32] and on motivational drives related to the safeguard of the social identity [33].
